# Twenty-Five Years of Evolution and Hurdles in Electronic Health Records and Interoperability in Medical Research: Comprehensive Review

**DOI:** 10.2196/59024

**Published:** 2025-01-09

**Authors:** Yun Shen, Jiamin Yu, Jian Zhou, Gang Hu

**Affiliations:** 1 Chronic Disease Epidemiology Population and Public Health Pennington Biomedical Research Center Baton Rouge, LA United States; 2 Department of Endocrinology and Metabolism Shanghai Sixth People's Hospital Affiliated to Shanghai Jiao Tong University School of Medicine Shanghai China

**Keywords:** electronic health record, electronic medical record, medical research, interoperability, eHealth, systematic review, real-world evidence, artificial intelligence

## Abstract

**Background:**

Electronic health records (EHRs) facilitate the accessibility and sharing of patient data among various health care providers, contributing to more coordinated and efficient care.

**Objective:**

This study aimed to summarize the evolution of secondary use of EHRs and their interoperability in medical research over the past 25 years.

**Methods:**

We conducted an extensive literature search in the PubMed, Scopus, and Web of Science databases using the keywords *Electronic health record* and *Electronic medical record* in the title or abstract and *Medical research* in all fields from 2000 to 2024. Specific terms were applied to different time periods.

**Results:**

The review yielded 2212 studies, all of which were then screened and processed in a structured manner. Of these 2212 studies, 2102 (93.03%) were included in the review analysis, of which 1079 (51.33%) studies were from 2000 to 2009, 582 (27.69%) were from 2010 to 2019, 251 (11.94%) were from 2020 to 2023, and 190 (9.04%) were from 2024.

**Conclusions:**

The evolution of EHRs marks an important milestone in health care’s journey toward integrating technology and medicine. From early documentation practices to the sophisticated use of artificial intelligence and big data analytics today, EHRs have become central to improving patient care, enhancing public health surveillance, and advancing medical research.

## Introduction

### Tracing Back to History

The practice of documenting patient information dates back approximately 3000 years, when early humans created some of the first known medical records by inscribing case histories on materials such as papyrus, clay tablets, animal bones, and other surfaces [1-3]. These records often included symptoms, diagnoses, and treatments. The health care delivery system developed with a long history and became increasingly complex. During the time of classical medicine, health care providers frequently gathered and systematized medical knowledge, creating standardized textbooks, which were often recorded by case series rather than individual patients [4]. In the 19th century, health care providers began keeping detailed paper records. These were used to track patients’ medical histories, nursing information, treatments, and outcomes. Hospitals and clinics maintained handwritten notes, which were stored in file systems [5-8]. With the explosion of electronic technologies boosted in the 20th century, hospitals began using such technologies to store records, reducing physical storage space and improving record retrieval efficiency. Computerization is one of the revolutionary technologies. The concept of electronic health records (EHRs) originated in the 1960s [9]. It aimed to improve the storage, retrieval, and management of patient information. Health care professionals started to address clinical challenges and steer treatment decisions by using data from these systems. Later, the 1990s brought about efforts to standardize EHR systems and the introduction of the internet, which significantly influenced EHR development. The 2010s witnessed widespread adoption of EHRs, driven by government incentives and the recognition of their potential to improve health care quality, safety, and efficiency [10-13]. Now, in the 2020s, the use of EHRs has been boosted with the development of advanced technologies in health care.

### Objectives

In the past 25 years, the scope of EHRs varied significantly across the globe. Initially, many EHR systems were developed primarily as billing tools and were not intended to support clinical workflows. However, over time, their functionality expanded to optimize diagnosis and patient care, thereby enhancing their utility and relevance for clinical research. This evolution has transformed EHRs into a valuable resource not only for managing health care delivery but also for facilitating data-driven research efforts. For the 25th anniversary of the *Journal of Medical Internet Research*, this review focused on the evolution of secondary use of EHRs and their interoperability in medical research over the past 25 years. For researchers using EHR data in medical studies, we explored the advancements in EHR technology, emphasizing how they have facilitated better management and understanding of diseases through comprehensive data collection and analysis. In addition, this review highlighted the importance of these records in epidemiological studies, pragmatic clinical trials (PCTs), and health economic studies, particularly in providing a more accurate representation of clinical practices and patient populations.

## Methods

### Literature Review

We focused on the advancements in EHR technology and the application and significance of these records in medical research across 3 distinct periods. Therefore, we conducted an extensive literature search in the PubMed, Scopus, and Web of Science databases, which provides ideal coverage of the publications of interest. We followed the PRISMA (Preferred Reporting Items for Systematic Reviews and Meta-Analyses) guidelines (Multimedia Appendix 1).

**Figure 1:**
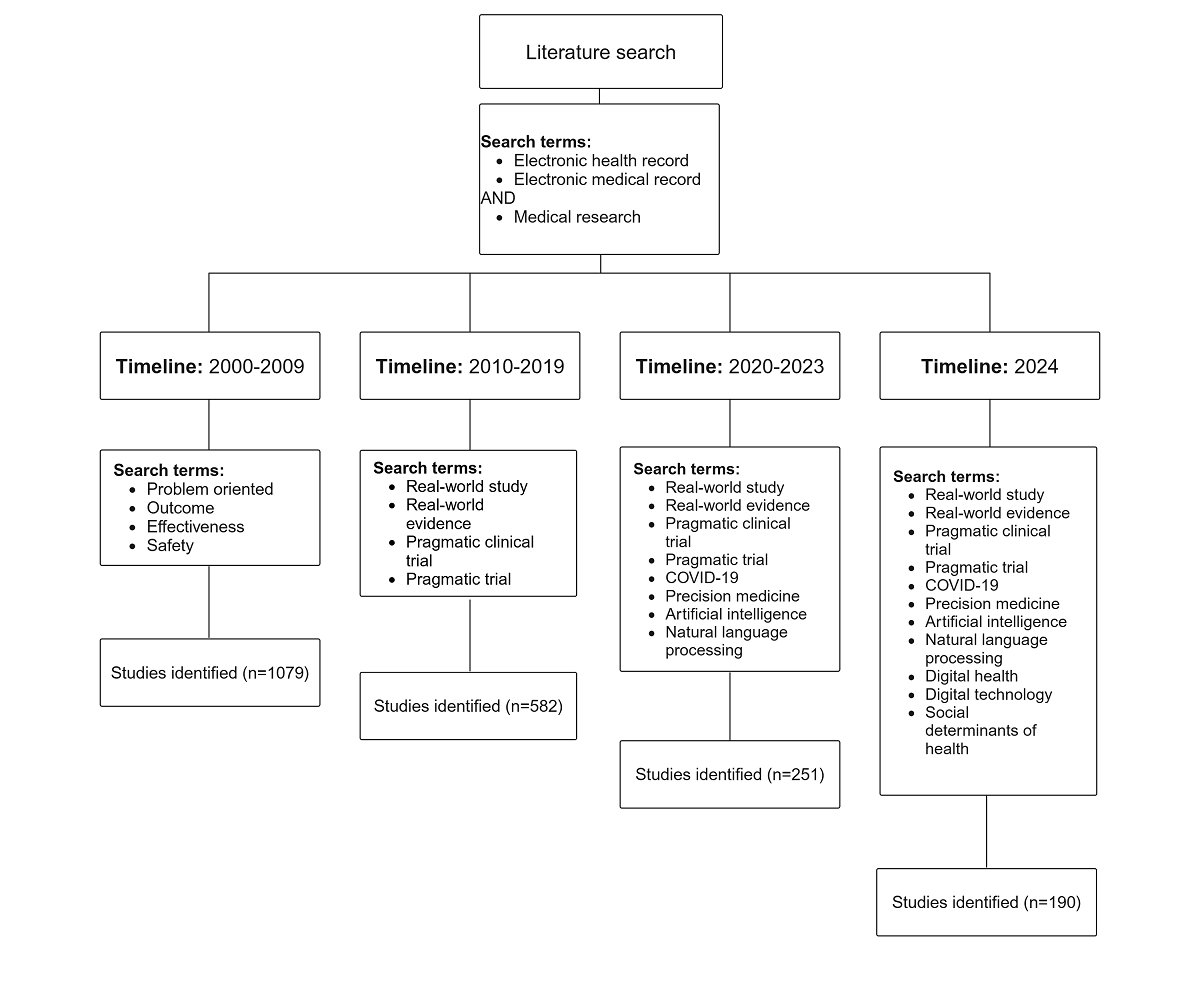
Search strategy and results by timeline.

Our search strategy was divided into three parts: (1) all studies that contained the terms *Electronic health record* or *Electronic medical record* in the title or abstract and *medical research* in all fields were identified; (2) according to the time of publication, the studies were divided into 2000s, 2010s, 2020 to 2023, and 2024; and (3) the studies were then narrowed down to fields by specific terms (Figure 1). For example, the search term for the 2010s was as follows: ((*electronic health record*[Title/Abstract]) OR (*electronic medical record*[Title/Abstract])) AND (*medical research*) AND ((*real world study*) OR (*real world evidence*) OR (*pragmatic clinical trial*) OR (*pragmatic trial*)). We used cross-referencing techniques from the included studies and supplemented our search with forward and backward citation tracking to ensure comprehensive coverage.

### Qualitative Structured Analysis

Qualitative analysis and structuring of the available sources were performed using NVivo (version 14; Lumivero) software. All studies were coded using NVivo to generate keywords.

## Results

### Eligible Studies

**Figure 2:**
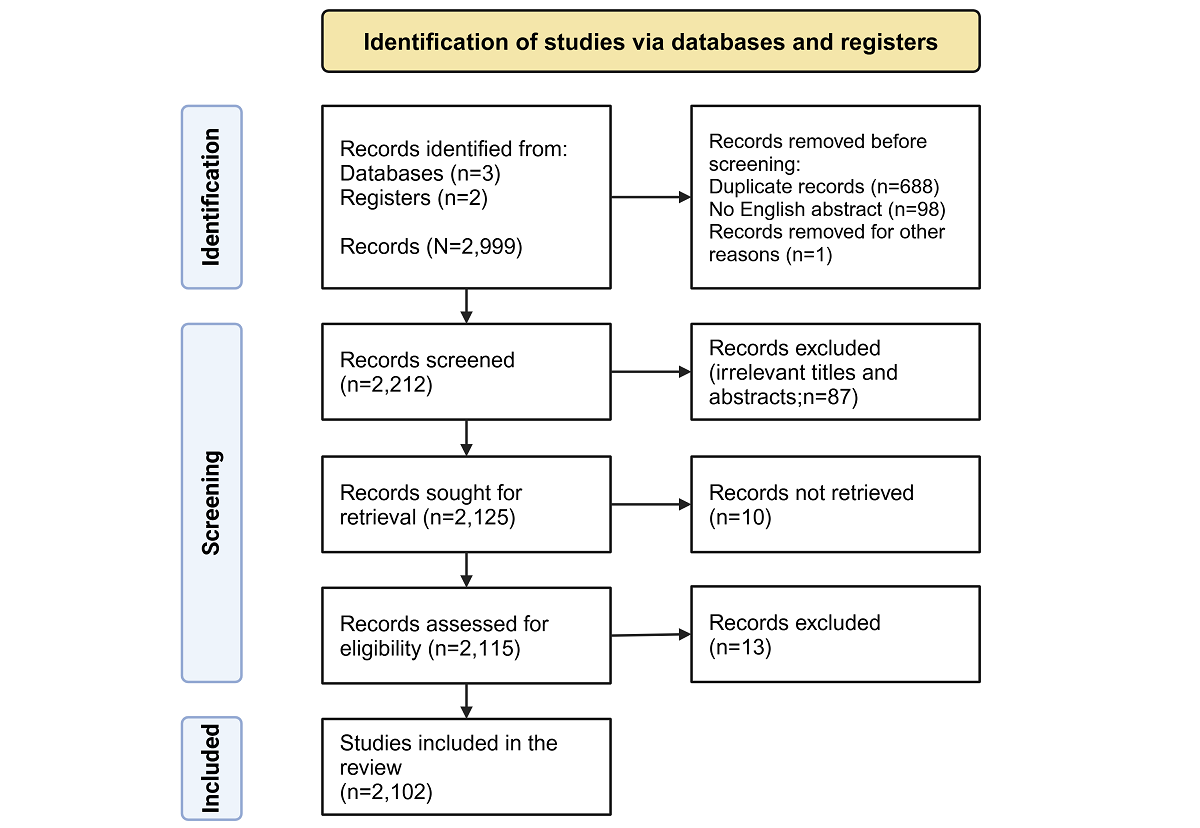
PRISMA (Preferred Reporting Items for Systematic Reviews and Meta-Analyses) flowchart.

According to the PRISMA framework (Figure 2), articles were searched in PubMed, Scopus, and Web of Science databases and categorized by period. Duplicate records, non-English abstracts, and records unrelated to EHRs were excluded, resulting in a final inclusion of 2102 articles. We also excluded records not pertinent to the application of EHRs in medical research, such as commentary articles and those focused on fraudulent claims.

### Time Intervals

The included articles highlighted the evolution of EHRs from simple documentation tools to central components of patient care, public health surveillance, and medical research. During the 2000s, researchers used EHRs in various observational studies and health care quality improvement efforts. During this period, early research using EHRs often focused on single health care systems with limited clinical data. The introduction of digital imaging and communications in medicine enabled the use of biomedical image data within EHRs, facilitating clinical follow-ups and case studies. During the 2010s, the EHRs served as primary data sources for many large-scale studies on patient outcomes, disease patterns, and treatment efficacy. Various national registries and research networks emerged to explore disease associations, health risks, and health care interventions. The concept of real-world evidence (RWE) gained attention, with PCTs relying heavily on EHR data to evaluate interventions in real-world settings. In addition, claim data were linked to EHRs for assessing health care services, resource use, and costs. Regulatory agencies began considering RWE in drug approvals and postmarketing surveillance. During the 2020s, retrospective EHR reviews enabled early identification of clinical characteristics, risk factors, and effective interventions, particularly focusing on the global health challenges of COVID-19 pandemic. Comparative effectiveness research using EHR data provided insights into public health measures, testing strategies, and treatment protocols. Globally, countries used their EHR infrastructure for real-time surveillance, vaccine distribution, and health policy adjustments. In addition, EHR data highlighted the impact of social determinants of health (SDOH) on COVID-19 outcomes. In addition, EHRs have played a pivotal role in precision medicine. Artificial intelligence (AI) and natural language processing (NLP) have increasingly been applied to EHRs, enabling predictive analytics for patient care, personalized treatments, and health care operations optimization. Many studies also highlighted the remaining limitations and challenges, including data quality, standardization, interoperability, patient privacy, data security, and high implementation costs. Enhanced regulatory frameworks, data-sharing agreements, and advanced security technologies were recommended to address these issues.

## Discussion

### Initial Adoption and Early Research Use Before and in the 2000s

#### The Problem-Oriented Medical Record

One of the earliest known efforts to use electronic systems for medical research that could be considered a precursor to modern EHRs was by Weed in 1968 [14]. Weed [14] introduced the concept of the problem-oriented medical record (POMR), which organized patient data according to specific problems, making it more systematic and usable for both care and research. Researchers began to use POMR for cost analysis [15], staff communications [16], and decision-making [17-19] in a wide range of diseases. The POMR was also strongly advocated among medical students for training purposes [20]. As patient-centered medical care always necessitates a patient-centered medical record, the POMR remained prevalent in the early 2000s [21-23].

The Regenstrief Institute, established in 1969 in Indianapolis, Indiana, is another pioneer in this field. The institute developed the Regenstrief Medical Record System (RMRS), one of the first electronic medical record systems [24,25]. The RMRS allowed for the electronic capture, storage, and retrieval of patient data, serving as a model for later EHR systems. Researchers began to design randomized trials by using the system to test the effectiveness of medications [26,27]. The RMRS attempted to create a community-wide linkage across health care systems, urgent care hospitals, clinics, and health departments [28]. Both Weed’s [14] work on POMR and the Regenstrief Institute’s development of the RMRS played significant roles in the early use of electronic systems to support medical research, laying the groundwork for the EHR systems we see today.

#### Technological and Methodological Developments

With the development of POMR and RMRS, some new technologies and methodologies were applied to support the use of EHRs in research. This included the creation of data warehouses and the development of tools for data mining and analysis, which would later become crucial for extracting meaningful insights from large EHR datasets [29,30]. The recognition of the value of EHR data for research led to the formation of consortia and collaborative networks aimed at sharing data and best practices. These early collaborations laid the foundation for later, more extensive networks that would leverage EHR data for multicenter studies and nationwide research initiatives [31].

In 2009, the Health Information Technology for Economic and Clinical Health Act (HITECH) was passed [12]. The HITECH Act represented a critical step in the digital transformation of the US health care system. The HITECH Act led to a substantial increase in the collection and digitization of patient health information. This vast repository of data became a valuable resource for researchers, offering a wealth of information on patient outcomes, disease patterns, and treatment effectiveness [32-36]. Most research studies in 2000s leveraging EHRs have some features in common, including a single health care system or single hospital, an observational study design, and limited data on clinical characteristics [37]. In addition, some researchers also tried to use EHRs for pharmacovigilance and comparative effectiveness of drugs [38-41]. The limited duration of interventional and observational periods in clinical trials necessitated real-time monitoring of therapeutic effectiveness and safety, crucial for both physicians and patients. The use of EHRs effectively addressed these concerns [42]. Some early explorations on biomedical image data began with the incorporation of the digital imaging and communications in medicine within EHRs [43-46]. The deidentification of radiology clinical data allowed researchers to conduct case series studies and to follow up the changing patterns of the biomedical images in a certain disease area.

### The Rise of Big Data in Health Care in the 2010s

In the 2010s, big data analytics in health care were popular, with EHRs serving as the primary data source. Researchers began leveraging EHR data to conduct large-scale studies on patient outcomes, disease patterns, and treatment efficacy. A lot of cohort studies have been designed and conducted using EHR data to provide evidence of the association between risk factors and occurrence as well as the prognosis of diseases. In addition, a lot of quality improvement studies used EHRs to investigate the patients’ communication and clinical support. The disease areas covered almost every system in the body, including primary care [47], cardiology [48], sepsis [49], cancer [50], diabetes [51], rheumatology [52], nephrology [53], neurology [54], hepatology [55], and gastroenterology [56]. Our previous review has highlighted the practical use of EHRs among patients with diabetes in medical research [57]. Moreover, insurance claim data, such as that from the Kaiser Permanente [58-60], offer valuable insights for enhancing our understanding of health risks, improving patient outcomes, and informing policy and practice decisions.

In contrast, researchers also started to analyze data from large patient populations to identify health trends, assess risk factors, and develop strategies for disease prevention and health promotion. They started to use these data for potential patient selections and recruitment for clinical trials [61]. Meanwhile, PCTs began to rise gradually [62]. Researchers have made significant efforts to bridge the gap between clinical trial results and real-world outcomes [63-66]. These type of trials were designed to evaluate the effectiveness of interventions or medications in real-world clinical settings, aiming to inform health care decision makers about the trade-offs between benefits, harms, and costs of those interventions [67-69]. They mainly rely on the EHR data. Researchers aimed at addressing key uncertainties in real-world clinical settings, focusing on comparing interventions or medications in ways that reflect usual care [70,71]. There were also some tools that were developed to help researchers design and implement PCTs, including the Pragmatic–Explanatory Continuum Indicator Summary (PRECIS-2) [72]. Moreover, stakeholder engagement is a pivotal step [73,74]. The implementation phase requires selecting appropriate sites that mirror the settings where the intervention will be applied, using strategies for diverse participant recruitment, and integrating interventions and comparators seamlessly into routine clinical practice. Data collection leverages EHR to minimize disruption and enhance the efficiency of the trial process. PCTs seem more reliable to provide RWE than traditional randomized trials. Some claim data linked to EHRs can also provide detailed records of health care services received by patients, allowing researchers to accurately measure resource use and associated costs [75-77]. This information is crucial for cost-effectiveness analyses that compare the costs and outcomes of different health care interventions. EHR data enable the estimation of direct medical costs by documenting clinical procedures, medication prescriptions, hospital stays, and other health care services. In addition, EHR data have contributed to the evaluations of health care quality and system performance, which supported its use in health policy studies, as the data contain a huge amount of information on health care procedures [78-80].

Regulatory agencies, including the US Food and Drug Administration (FDA) [81] and the European Medicines Agency (EMA) [82], began to explore the potential of RWE from real-world studies of large cohorts or PCTs in supporting drug approvals and postmarketing surveillance. The Patient-Centered Outcomes Research Institute in the United States, which was established in 2010, also reflected a growing recognition of the value of RWE in regulatory decisions and health care policy [83,84]. The FDA and EMA considered approval seriously with evidence from RWE, particularly when traditional clinical trials are not feasible or practical [85]. This is particularly relevant in scenarios where patient populations are rare, heterogeneous, or difficult to recruit into randomized controlled trials. Regulatory agencies, including the FDA and the EMA, have increasingly recognized the value of RWE in regulatory decision-making. The FDA’s 21st Century Cures Act has encouraged the use of RWE in supporting the approval of new indications for existing drugs and in fulfilling postmarketing study requirements [85]. A notable example of this is the expanded indication of palbociclib, which was approved by the FDA for use in male patients with hormone receptor–positive, HER2-negative advanced or metastatic breast cancer [86]. In 2020, the FDA approved pembrolizumab for the treatment of adult and pediatric patients with unresectable or metastatic solid tumors that have a high tumor mutational burden [87]. This decision involved RWE derived from genetic sequencing and EHRs. Other examples include the approval of blinatumomab for pediatric acute lymphoblastic leukemia [88], ibrutinib for chronic lymphocytic leukemia [89], nusinersen for spinal muscular atrophy [90], and alosetron for irritable bowel syndrome in female patients [91].

### Global Health, Precision Medicine, and AI in the 2020s

#### COVID-19 Pandemic and Real-Time Surveillance

The global nature of the COVID-19 pandemic increased the importance of international collaboration in medical research. While the health care providers were busy taking actions against the COVID-19 pandemic, they used retrospective EHR to learn lessons and analyze the trends and responses they had made [92-98]. The World Health Organization declared the outbreak a public health emergency of international concern (PHEIC) in early 2020 [99] and ended the PHEIC declaration in 2023 [100]. During the early stages of the COVID-19 pandemic in 2020, Chinese physician-scientists played a crucial role in understanding the disease by conducting retrospective reviews of medical records [93,95-98]. This process was instrumental in identifying the clinical characteristics and transmission patterns of COVID-19 infection, at a time when the world was scrambling for information on how to deal with the novel coronavirus. Importantly, these studies highlighted the disease’s high transmissibility, both from symptomatic and asymptomatic carriers, underlining the importance of widespread testing, contact tracing, and quarantine measures to control its spread [101-106]. This group of physician-scientists from Wuhan, China, also contributed to the understanding of risk factors associated with severe outcomes, including age, pre-existing health conditions, and certain demographic factors. Their findings on the efficacy of various public health interventions, such as the use of face masks, social distancing, and the implementation of lockdowns, informed policies not just in China but around the world [107,108]. Furthermore, the early identification of the genome sequence of SARS-CoV-2 virus by researchers facilitated the rapid development of diagnostic tests and the initiation of vaccine development efforts [109-111]. This early sharing of data was pivotal in the global race to develop vaccines and therapeutics against COVID-19.

During the midpandemic period, EHR data became instrumental for comparative effectiveness research, aimed at evaluating and improving health outcomes across various populations and health care systems. This research spanned a broad spectrum of topics crucial to pandemic management, including the effectiveness of personal protective equipment [112-114], the impact of social distancing [115,116], the accuracy and efficiency of different testing strategies [117,118], strategies [117,118], the efficacy and safety of antiviral medications and vaccines [119-123], and the role of health supplements [124]. In addition, it explored the role of telemedicine in sustaining patient care and the importance of primary care services during the COVID-19 pandemic [115,125-128]. For example, comparative effectiveness research helped identify the types of personal protective equipment that were most effective in preventing virus transmission among health care workers and the public [129,130]. Similarly, studies on social distancing and lockdown measures offered evidence-based guidance on how to balance public health concerns with economic and social implications [131]. The investigation into testing strategies elucidated the strengths and weaknesses of various diagnostic approaches, leading to improvements in testing strategies and the quicker identification of COVID-19 cases [132]. Research on antiviral drugs, anti-inflammatory drugs, and some health supplements informed treatment protocols, contributing to the development of therapeutic strategies that reduced the severity and duration of the disease [133,134]. Telemedicine emerged as a vital tool for providing continuous care while minimizing the risk of virus transmission, demonstrating its potential to revolutionize health care delivery beyond the pandemic [135,136].

In addition to China, in the United States, EHR systems also played a crucial role in the national response to COVID-19. Through partnerships with health care systems and EHR vendors, the Centers for Disease Control and Prevention, the US FDA’s Sentinel System and other public health authorities gained access to near–real-time data on COVID-19 cases, hospitalizations, ventilator use, and patient outcomes [137]. EHRs were also instrumental in tracking vaccine distribution and administration. The Vaccine Adverse Event Reporting System used EHR data to monitor postvaccination side effects, guiding policy adjustments in vaccination campaigns [138]. In the United Kingdom, the government leveraged its National Health Service (NHS) EHR infrastructure for real-time surveillance and policy making during the COVID-19 pandemic. The United Kingdom developed the OpenSAFELY platform, a secure analytics environment that allowed researchers to access deidentified EHR data of >58 million NHS patients [139]. EHR data were used to identify individuals with high risk and provided real-time data on hospital admissions, intensive care unit (ICU) bed occupancy, and patient outcomes. South Korea used a robust real-time surveillance system that integrated EHRs with other health data sources to contain the spread of COVID-19, including contact tracing and national health reporting [140]. In Australia, the national My Health Record system was used to support the country’s pandemic response with centralized health data access and the implementation of lockdowns and the reopening of services [141]. Germany also used EHR data to manage its health care resources effectively during the COVID-19 pandemic, which used EHR data to monitor ICU capacity across the country. Hospitals provided daily updates on bed occupancy, ventilator availability, and staffing levels [142]. In addition, Germany explored the use of EHRs to develop digital health passports that recorded patients’ vaccination status and COVID-19 test results [143]. The use of EHRs for real-time surveillance and policy making during COVID-19 highlighted the importance of interoperable systems. Countries with centralized health records or integrated health information exchanges were better positioned to use EHR data quickly and effectively.

The COVID-19 pandemic has also accentuated the critical role of SDOH, revealing stark disparities in disease outcomes across different populations [144]. EHRs have provided a rich dataset for analyzing these disparities, uncovering the disproportionate burden of COVID-19 on specific groups such as the older adults, individuals with chronic diseases, and those with autoimmune diseases. Studies leveraging EHR data have identified various social determinants that contribute to the increased vulnerability of these populations. Factors such as socioeconomic status, access to health care, living conditions, and occupational hazards have been linked to higher rates of infection, severe disease outcomes, and mortality [145-151]. For instance, individuals in lower socioeconomic brackets often face barriers to accessing health care services, leading to delayed or inadequate treatment for COVID-19 [152]. Similarly, patients with pre-existing chronic conditions such as diabetes, cardiovascular diseases, and respiratory diseases have been shown to experience more severe outcomes when infected with the virus [105,153]. Furthermore, the research has highlighted the importance of addressing these social determinants in pandemic response strategies. Interventions targeting SDOH can help mitigate the adverse outcomes of COVID-19 among populations considered vulnerable. This included improving access to health care, enhancing support for individuals with chronic diseases, ensuring the availability of protective measures for individuals classified as immunocompromised, and addressing the broader socioeconomic and environmental factors that contribute to health disparities [154].

Even after the end of the PHEIC declaration, the use of EHR in studying post–COVID-19 condition continued to be of paramount importance. Research into the long-term impacts of COVID-19, particularly on mental health [155-158] and cognitive functions [159-161], remains a critical area of public health inquiry. EHRs were expected to provide invaluable, real-time data that can link early exposure to COVID-19 with outcomes that may manifest decades later or even across a lifetime. As humanity progresses, EHRs stand as a vital resource, equipping us to face global crises and unpredictable challenges with resilience and informed action.

#### Precision Medicine and Genomics

Precision medicine represents a transformative approach to health care in the past 10 years, focusing on the customization of treatment based on an individual’s genetic makeup, environment, and lifestyle [162]. EHRs played a pivotal role in the advancement of precision medicine, where treatments are tailored to the individual characteristics of each patient. The integration of genomics data with EHRs allowed researchers to study the genetic bases of diseases and to personalize health care at an unprecedented level. However, integration is still facing several challenges and opportunities. While the routine clinical application of genomic data is still emerging, some EHR-connected biobanks and initiatives are actively addressing the challenges involved. While the EHR-connected biobanks were initially designed for research purposes, it has actively promoted to incorporate the biobank data into the EHR systems.

The UK Biobank (UKB) stands as a prominent figure in the realm of biomedical research, featuring an expansive database and research resource. It encompasses deidentified genetic, lifestyle, and health information, along with biological samples from half a million UK participants [163]. This rich dataset is further augmented by linkage to the United Kingdom’s EHRs, such as the Hospital Episode Statistics and the Patient Episode Databases [164,165]. The UKB’s comprehensive dataset has been instrumental in propelling forward the fields of medicine and research, offering profound insights into the prevention, diagnosis, and treatment of numerous serious and life-threatening diseases. The UKB has released a set of whole genome sequencing data. Since 2018, numerous studies have used these data, along with Mendelian randomization, to investigate the causal relationships between genetic backgrounds and diseases [166-169]. While the UKB serves as a notable instance of linking a study cohort with EHRs, it does not exemplify direct integration. Despite encompassing data from half a million individuals, the UKB faces potential selection bias [170]. This is attributed to the fact that the initiative dispatched >9 million invitations across the nation, suggesting that the participants may not fully represent the broader population.

Similar to the approach of the UKB, the National Institutes of Health in the United States initiated the All of Us Research Program in 2018, aiming to enroll more than a million individuals [171]. By January 1, 2024, the program had successfully registered >760,000 participants, securing access to >539,000 samples for genomic sequencing and 420,000 EHRs. Through its comprehensive data collection, the All of Us Research Program seeks to address critical gaps in medical research, particularly in areas underserved by existing studies, thereby fostering a deeper understanding of various health conditions and improving strategies for prevention, diagnosis, and treatment across the spectrum of both common diseases and rare diseases. However, it remains a strong example of linking cohort data with EHRs.

Other local or nationwide biobanks with linkage to EHRs or national registries include the Danish Biobank Register [172]; BioBank Japan [173]; China Kadoorie Biobank [174]; Kaiser Permanente Research Bank [175]; deCODE Genetics [176]; FinnGen biobank [177]; Vanderbilt University’s bioVU [178]; Michigan Genomics Initiative [179]; BioMe biobank [180]; the Estonian biobank [181]; and the Norwegian Mother, Father and Child Cohort Study [182]. These initiatives varied in scope and scale but collectively contributed to the growing field of genomic research linked to EHRs, facilitating advances in personalized medicine and the understanding of genetic influences on various diseases.

The true sense of integrating genomic data into EHRs presented challenges such as diverse data structures, storage limitations, interpretation complexities, and financial constraints. The National Human Genome Research Institute supported the Electronic Medical Records and Genomics network to address these issues by harmonizing data and developing guidelines for genomic data integration and use [183]. Solutions are in progress, and the initiative now has >10 clinical site partners, along with a network of affiliates across major areas in the United States. The Electronic Medical Records and Genomics network is not only developing best practices for electronic phenotyping and biobanking consultation but also exploring EHRs for additional genome-wide association studies outcomes among genotyped individuals, leveraging the rich phenotypic data available.

#### Data Analytics and AI

The use of data analytics and AI in analyzing EHR data has grown exponentially. Researchers were using machine learning algorithms to identify patterns, predict outcomes, and generate insights into disease progression, treatment efficacy, and patient care optimization [184,185]. These technologies enabled the analysis of vast amounts of data in ways that were not previously possible.

#### Predictive Analytics for Identifying Patients With High Risk and Their Outcomes

One of the most significant applications of AI in health care is in identifying patients at high risk of developing specific conditions and predicting outcomes among patients who were already diagnosed with a health condition. By analyzing historical EHR data, including patient demographics, previous health conditions, treatment histories, and lifestyle factors, AI models can predict which patients are more likely to develop diseases such as diabetes, cardiovascular diseases, or chronic respiratory conditions, as well as the potential outcomes of various treatment options [186-190]. Early identification of these patients allows health care providers to implement preventive measures, tailor treatments to individual needs, and in doing so, significantly reduce the risk of severe outcomes or the need for hospitalization. This predictive capability enables physicians to choose the most effective treatment plans, thereby improving the quality of care and patient satisfaction. In the context of infectious diseases, AI models are invaluable for tracking and forecasting the spread of diseases. By integrating EHR data with information from other sources, such as public health records and social media, AI algorithms can predict outbreaks, model the spread of infections, and inform public health interventions [191,192]. This was particularly evident during the COVID-19 pandemic, where AI played a pivotal role in predicting hospitalization rates, identifying potential hot spots, and guiding the allocation of medical resources [193,194].

#### Personalized Medications and Clinical or Regulatory Decision Support Systems

Leveraging EHR data, AI is facilitating personalized medicine by tailoring treatment plans to individual patient characteristics, genetic information, and historical health data. This approach helps in predicting how different patients will respond to various treatments, thereby improving treatment effectiveness and reducing side effects [195]. While medications only serve one part of the disease management, AI integrated with EHR systems can provide clinicians with decision support by offering diagnostic suggestions, recommending treatment options, and alerting health care providers to potential adverse drug interactions or necessary screenings, thereby enhancing patient care quality [196-200]. AI tools also analyze EHR data to optimize hospital operations and predict perioperative complications. This can lead to improved patient satisfaction, lower surgery-related adverse events, and lower health care costs [201-205]. Among the AI strategies, NLP technologies represent a pivotal advancement in the use of EHR data, transforming the landscape of health care data analytics and decision-making [185]. NLP enables the extraction of valuable information from unstructured text within EHRs, such as clinical observations, patient histories, and diagnostic notes, which traditionally have been difficult to analyze systematically due to their unstructured nature. This capability not only enhances the richness of data available for clinical decision-making but also significantly expands the potential for personalized patient care and advanced health care analytics [206-209]. One notable example of NLP’s application in health care is the development of GatorTron (University of Florida), a large clinical language model trained on >90 billion words from clinical notes, PubMed articles, and Wikipedia [210-212]. GatorTron significantly outperforms previous models in clinical NLP tasks such as clinical concept extraction, medical relation extraction, semantic textual similarity, natural language inference, and medical question answering. This model showcases how scaling up the number of parameters and data size can lead to substantial improvements in performance across various NLP tasks, ultimately enhancing the extraction and interpretation of critical information from EMR data for better health care delivery and research.

#### EHRs in Medical Research in the Next Decades

**Figure 3:**
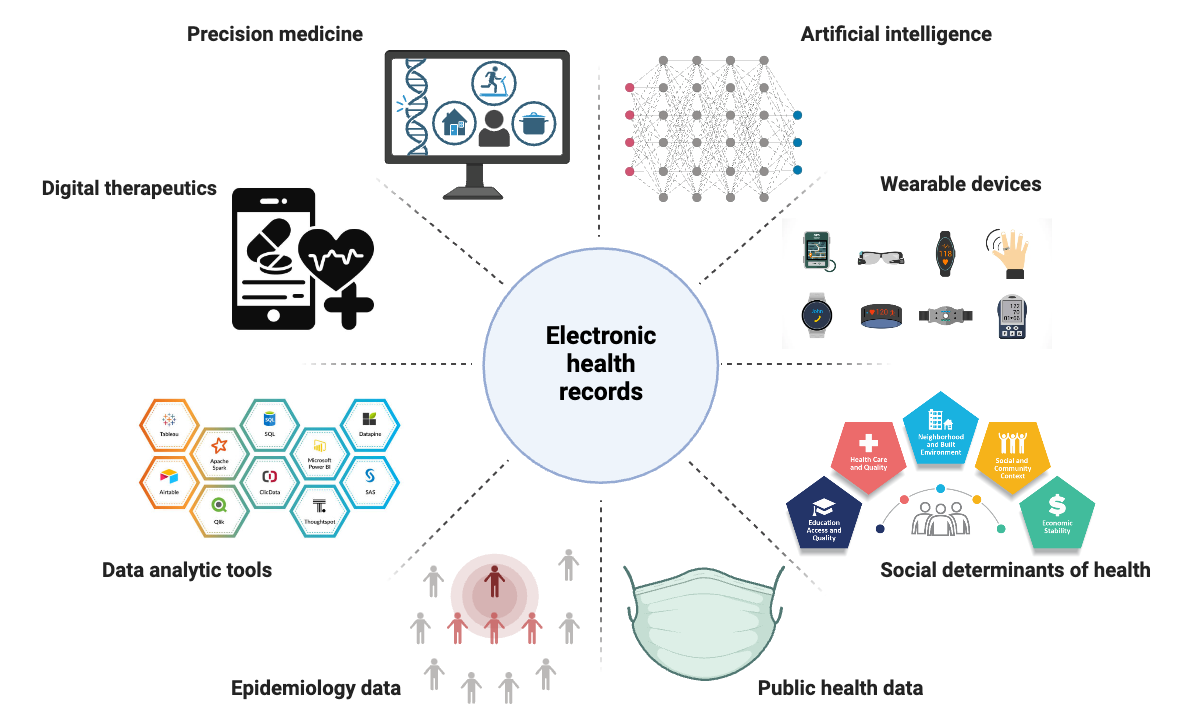
Future landscape of electronic health records.

The next decades will likely see significant advancements in EHR capabilities, focusing on interoperability, precision medicine, AI and digital health technologies, the integration of SDOH, public health monitoring, and the enhancement of data analytic tools (Figure 3).

#### EHRs for Patient Care Versus EHRs for Research

It has been a trend to develop an EHR system for research use only. EHRs designed for patient care and those designed for research may differ in their structure, focus, and functionality [213]. EHRs tailored for patient care will prioritize real-time clinical decision support, patient safety, and continuity of care. They are built to store detailed patient information, such as medical history, medications, allergies, laboratory results, and diagnostic imaging, allowing health care providers to quickly access and update data during clinical encounters. The interface is designed for ease of use. In contrast, EHRs designed for research will focus on data collection, aggregation, and analysis at a population level. They emphasize data standardization, coding, and interoperability, allowing researchers to extract large datasets for epidemiological studies, clinical trials, and public health surveillance [214]. These EHRs will include extensive metadata and use structured data formats to facilitate the analysis of trends and outcomes. While patient care EHRs focus on the individual patient, research-oriented EHRs aggregate deidentified data from diverse populations to explore disease patterns, treatment effectiveness, and health care disparities. In addition, research EHRs must also address data privacy and consent issues more rigorously to comply with ethical standards for data use [214].

#### Enhanced Interoperability and Data Sharing

Future EHR systems are likely to achieve higher levels of interoperability, enabling seamless data sharing across different health care systems, health care providers, and even countries. This will facilitate comprehensive patient care, collaborative research, and public health monitoring on a national or even a global scale. Regulations governing the use of EHRs and related technologies will evolve to keep pace with advancements, addressing ethical considerations, data ownership, and the use of digital health data in research and health care delivery. As the use of EHRs grows, the focus on protecting patient data will also be important. Advanced security technologies and privacy-preserving methods, such as blockchain and federated learning, may be used to safeguard information while enabling its use for care and research.

As a good example, the CODE-EHR framework represents a crucial advancement, focusing on improving the design of studies that use structured EHR records [215]. This framework emphasizes the importance of verification, validation, and data privacy, along with addressing social mandates to undertake research. It proposes minimum standards to enhance study transparency and provides a road map toward more effective use of health care data for research. By enabling the embedding of controlled trials within real-world settings, such as registries or routine clinical practice, CODE-EHR aims to produce more generalizable results that can inform patient care, health care cost containment, and quality of life improvements.

#### Integration of Genomic and Other Omics-Related Precision Medicine Data

As genomic sequencing becomes more affordable and commonplace, EHRs are expected to integrate multiomics information, supporting the advancement of precision medicine. This will allow health care providers to tailor treatments to individual patients based on their individualized profiles, improving outcomes and minimizing adverse reactions [216].

The PennChart Genomics Initiative has demonstrated significant progress and potential in integrating genomic data into EHRs for optimizing patient care and facilitating medical research [217,218]. By creating a centralized document display for genetic results in the EHR, researchers and clinicians can improve patient care while addressing privacy concerns. This initiative has already led to the integration of thousands of genetic documents into the EHR, highlighting the potential for EHRs to support precision medicine and accelerate the discovery of novel genomic medicine applications. Future efforts aim to expand this integration and address challenges such as educational needs, infrastructure compatibility, and maintaining privacy and security protections.

The Genetics Underlying Diabetes in Hispanics and Latinos study in the United States is a pioneering initiative planning to enroll 100,000 newborns for whole genome sequencing [219]. This program aims to explore the potential of genomic data in newborn screening, with the goal of enhancing early diagnosis and treatment in neonatal ICUs and pediatric ICUs. By identifying genomic markers early on, the program seeks to save lives and reduce hospital stays for patients with undiagnosed disease, showcasing the transformative power of genomics in precision health care.

Genomics England’s program is another potential effort to integrate genetic data with EHR data, aiming to sequence 100,000 genomes from NHS patients with rare diseases and their families as well as patients with cancer [220]. Launched to enhance our understanding of genetics in disease and to pioneer a UK genomic medicine service, the project’s success has paved the way for further genomic research and integration into health care. This initiative highlights the potential of genomic data in advancing personalized medicine and improving treatment strategies.

#### Expansion of AI and Other Digital Health Technologies

EHRs in the future will increasingly incorporate AI and machine learning algorithms to analyze health data in real time, predicting patient risks (such as for chronic diseases or hospital readmissions) and suggesting personalized prevention and treatment plans. AI-driven models, using big data from EHRs, outperform traditional methods in sensitivity, specificity, and other metrics. The implementation of these models into web applications and mobile apps could significantly aid clinical decision-making, emphasizing the need for effective strategies to integrate AI algorithms in clinical tools.

The future use of NLP in EHRs is also expected to significantly enhance health care delivery and research [221]. By leveraging NLP, health care professionals can continuously extract valuable insights from the increasing volumes of unstructured data within EHRs. This advancement will allow for more accurate patient stratification, personalized treatment plans, and improved predictive models for disease outcomes in the future. NLP technologies are set to automate the extraction and interpretation of clinical information, reducing manual data entry and analysis burdens. In addition, real-time processing of patient data through NLP will facilitate more rapid clinical decision support, optimizing treatment approaches and patient care pathways [222]. The integration of NLP with EHR systems promises a shift toward more data-driven, efficient, and patient-centered health care, highlighting its critical role in advancing medical research, enhancing disease surveillance, and ultimately improving patient outcomes.

In addition, EHRs will also become more integrated with other digital health technologies, such as wearable devices, telehealth platforms, and digital therapeutics [223,224]. Wearable devices, ranging from fitness trackers to advanced biosensors, have become increasingly sophisticated, capable of monitoring a wide array of health metrics such as heart rate, activity levels, sleep patterns, and blood glucose levels. Integrating these devices with EHRs can provide clinicians with a more comprehensive view of a patient’s health status, extending beyond the snapshots captured during clinical visits. This continuous stream of real-time data can enhance preventive care strategies, enable early detection of potential health issues, and support personalized treatment plans. For patients with chronic conditions, this integration can facilitate closer monitoring and adjustments to treatment regimens based on near–real-time data, potentially improving outcomes and patient engagement in their care. Telehealth platforms have experienced exponential growth, a trend significantly accelerated by the COVID-19 pandemic. The integration of telehealth with EHRs ensures that online consultations are informed by the patient’s medical history, current medications, and recent test results, allowing for more informed clinical decision-making [225]. This seamless flow of information can improve the efficiency and effectiveness of virtual care, making health care more accessible, particularly for individuals in remote or underserved regions. Furthermore, integrating telehealth encounters into EHRs ensures that all aspects of a patient’s care are documented in a single, comprehensive record, supporting continuity of care across different health care settings. Digital therapeutics, which include software-driven evidence-based interventions, are an emerging field within health care [226]. These interventions, which can range from cognitive behavioral therapy apps for mental health to digital programs for chronic disease management, offer new avenues for treatment that are accessible and scalable. Integrating digital therapeutics with EHRs can enhance the personalization of care, enabling health care providers to track patients’ engagement and progress with these interventions in parallel with traditional treatments. This integration can also facilitate the collection of outcome data, supporting the evaluation of the effectiveness of digital therapeutics in real-world settings and informing future care decisions.

#### Incorporation of SDOH

Addressing SDOH is crucial for improving health outcomes and reducing health disparities. Future EHR systems may include more comprehensive data on patients’ social, economic, and environmental conditions, which are currently lacking in most systems and platforms [227]. This idea of data integration will empower health care providers to address not just the clinical but also the contextual aspects of health, leading to more targeted interventions, improved health outcomes, and reduced health care disparities. The evolution of EHR systems to include comprehensive SDOH data will necessitate advancements in data collection, privacy protocols, and analytics. Collecting SDOH data in EHRs in the future could involve leveraging technology to automate data capture from various sources, including patient self-reporting through digital platforms, integrating community health data, and using NLP to extract SDOH information from clinical narratives.

#### Future Use in Public Health and Epidemiology

EHRs will continuously play a vital role in monitoring public health trends due to their performance during the COVID-19 pandemic, managing outbreaks of infectious diseases, and conducting large-scale epidemiological studies in the future in case of other unpredictable pandemics. Real-time data analysis will enable quicker responses to public health emergencies and more effective preventive strategies. However, these potential opportunities will depend mainly on the enhanced interoperability and data sharing, as mentioned previously.

#### Integration of Data Analytic Tools With EHRs

Integrating data analytics tools with EHRs involves connecting the capabilities of data analysis software with the wealth of patient information stored in EHR systems. This integration can enhance health care delivery, improve patient outcomes, and facilitate health care research by leveraging advanced analytics on EHR data. A recent effort for an integrated online phenomics knowledge base within the EHR data was the development of the Centralized Interactive Phenomics Resource (CIPHER) knowledge base [228,229]. The CIPHER is a comprehensive, public-facing knowledge base designed to streamline the development of clinical phenotypes, aiming at facilitating clinical and health services research. It also featured scalable metadata management, integrated tools, and user workflows as well as enabled complex searches through stored phenotype metadata adhering to the CIPHER standard. Phenotypes can be contributed via a webform that validates metadata, and the platform includes data visualization tools to improve user engagement and accelerate phenotype development. Hopefully, the CIPHER knowledge base can expand the phenotype algorithm repository and the collaborations among users in the future.

### Potential Challenges

It also highlighted several challenges in using EHRs for medical research before and in the past 25 years, including issues with data quality, consistency, and interoperability between different EHR systems. Privacy and security concerns regarding the use of patient data for research purposes were significant, leading to regulatory efforts to protect patient information while enabling research. The Health Insurance Portability and Accountability Act (HIPAA), originally enacted in 1996, continued to influence EHR development throughout the 2000s with its privacy and security rules, which were critical in shaping how EHR systems handled sensitive patient data [230,231].

### Deidentification, Recoding, Regulatory, and Ethical Considerations

In the clinical care setting, information is recorded into EHRs by various health care professionals, including physicians, nurses, medical assistants, and other clinical staff. The accuracy and completeness of the data entered depend heavily on the documentation practices of these health care providers, which can vary between institutions and countries [232]. In some cases, administrative staff may also input data related to billing and patient demographics. It is important to note that these data inputs are primarily geared toward patient management rather than research, which introduces variations and challenges when using EHR data for secondary research purposes. One of the most crucial steps in preparing EHR data for research is deidentification, which involves removing or masking identifiable patient information (eg, names, addresses, and social security numbers) to protect patient privacy. Data security and privacy concerns often influence how EHR data are accessed and used for research. Institutions often have governance committees or data access boards that review research proposals to ensure they comply with ethical guidelines and data use policies. However, these regulations vary from country to country and system to system. In the United States, HIPAA regulations define strict guidelines for deidentification and the secondary use of health data. Institutions may use institutional review boards to oversee the approval process for research using EHR data [230]. In the European Union, under General Data Protection Regulation, pseudonymization and data minimization are encouraged, and the use of EHR data for research typically requires explicit patient consent unless an exemption is granted [233]. In the United Kingdom, the NHS has implemented the use of trusted research environments to facilitate secure access to health data for research while ensuring patient privacy [234].

Given the differences in regulations across countries, researchers must familiarize themselves with the specific legal and ethical requirements of the country where the EHR data are stored and where the research will be conducted. When conducting international or multicountry research, researchers need to consider how differing regulations affect data sharing, processing, and consent. Cross-border collaboration may require legal agreements, such as data transfer agreements, that meet all involved jurisdictions’ requirements. Regardless of the region, maintaining high standards for data security and privacy is crucial. Researchers should use practices such as data pseudonymization, anonymization, encryption, and secure data storage to protect patient information throughout the research process [235].

Despite the progress, researchers continued to face challenges with the interoperability of EHR systems. The ability to share and integrate data across different platforms and health care institutions was still limited, posing hurdles to multisite studies and collaborative research efforts. Addressing these challenges became a priority for advancing the use of EHRs in research. As the use of EHR data in research grew, concerns about patient privacy and data security also increased. Increased attention to regulatory and ethical issues surrounding the use of health information came up. Policies and guidelines, such as the HIPAA in the United States and the General Data Protection Regulation in the European Union [233,236,237], were updated to ensure the protection of patient information while facilitating research. Despite these concerns, all the efforts during this time underscore a shift toward a more holistic approach to evidence generation, aiming to improve patient outcomes and health care decision-making by bridging the gap between research such as clinical trials and real-world practice.

Meanwhile, researchers also encountered challenges during the use of EHRs on the common data models and data cleaning complexities. Although the Patient-Centered Outcomes Research Institute has made great efforts to this issue by establishing the PCORnet common data model [238-240] and the Observational Health Data Sciences and Informatics consortium has developed the Observational Medical Outcomes Partnership Common Data Model [241-243], significant challenges remain in achieving interoperability between different EHR systems and health care databases. This inconsistency can hinder the seamless exchange and integration of health data for comprehensive analyses. Common data models rely on the assumption that data across systems can be standardized into a uniform format. However, variations in how data are recorded, missing information, and inconsistent use of terminologies across different EHR systems can lead to gaps and inconsistencies that affect data quality and utility. For the concerns of data cleaning and linkage, although several standardized coding systems (ie, International Classification of Diseases, Current Procedural Terminology, Healthcare Common Procedure Coding System, Logical Observation Identifiers Names and Codes, Systematized Nomenclature of Medicine, etc] were introduced in the EHRs, they still contain a vast amount of unstructured or semistructured data, including free-text clinical notes [244]. Cleaning such data to extract meaningful information may require other tools and algorithms, such as SQL environment [MySQL version 8.4.0; Oracle Corporation] and Python [version 3.8.5; Python Software Foundation]), which can be resource intensive and complex to develop and apply for researchers without computer science backgrounds. Patients’ data are often shared among different EHR systems, including primary care and specialty registries. Linking these fragmented data sources to achieve a holistic view of a patient’s health history is challenging but essential for effective research and care management.

### Data Quality, Standardization, Patient Engagement, Cost, Data Cleaning, and Global Health Disparities

Ensuring high-quality, standardized data entry into EHRs is challenging yet essential for effective health care delivery and research. Inconsistent data, incomplete records, and variation in data capture practices can compromise the reliability and usefulness of EHR data for clinical decision-making and research purposes. While patient portals and digital access to health records aim to empower patients, engaging patients effectively remains a challenge. Issues such as digital literacy, accessibility, and varying levels of interest in active health management can affect the extent to which patients use EHRs to participate in their care [245].

For many health care providers, particularly smaller practices and those in low-resource settings, the cost of EHR implementation and ongoing maintenance is a significant barrier [246,247]. In addition, the process of transitioning from paper-based systems to EHRs, or switching between EHR systems, can be disruptive and resource intensive. Global disparities in EHR adoption and use highlight the challenge of ensuring equitable access to the benefits of digital health records. Differences in infrastructure, funding, and health care policies contribute to varying levels of EHR integration and use worldwide. Integrating AI and advanced analytics into EHR systems presents both opportunities and challenges. While these technologies have the potential to transform health care delivery and research, they also raise questions about data accuracy, algorithmic bias, and the interpretability of AI-generated insights.

### Limitations

Despite extensive literature searches across three databases and rigorous literature screening processes, our systematic review still faces some limitations. First, publication bias remains a concern, as studies reporting positive or significant findings are more likely to be published and thus overrepresented in our review. In addition, studies on EHR are highly heterogeneous. Variations in study design, populations, interventions, outcomes, and methodologies complicate meaningful synthesis and generalization of findings. Finally, some relevant studies may be unpublished, inaccessible, or have incomplete data, resulting in restricting data collection and limiting the overall comprehensiveness of our review.

### Conclusions

The evolution of EHRs marks a significant milestone in health care’s journey toward integrating technology and medicine. From early documentation practices to the sophisticated use of AI and big data analytics today, EHRs have become central to improving patient care, enhancing public health surveillance, and advancing medical research. As we look forward, the integration of SDOH, genomic data, and real-time analytics into EHRs promises to further personalize medicine, improve disease prevention strategies, and address health disparities. Collaborative efforts across borders, disciplines, and sectors will be crucial in realizing the full potential of EHRs in shaping a healthier future for all.

## Appendix

Multimedia Appendix 1 PRISMA (Preferred Reporting Items for Systematic Reviews and Meta-Analyses) checklist.

## Abbreviations

AI: artificial intelligence
CIPHER: Centralized Interactive Phenomics Resource
EHR: electronic health record
EMA: European Medicines Agency
FDA: Food and Drug Administration
HIPAA: Health Insurance Portability and Accountability Act
HITECH: Health Information Technology for Economic and Clinical Health Act
ICU: intensive care unit
NHS: National Health Service
NLP: natural language processing
PCT: pragmatic clinical trial
PHEIC: public health emergency of international concern
POMR: problem-oriented medical record
PRISMA: Preferred Reporting Items for Systematic Reviews and Meta-Analyses
RMRS: Regenstrief Medical Record System
RWE: real-world evidence
SDOH: social determinants of health
UKB: UK Biobank
